# Terpene Synthases as Metabolic Gatekeepers in the Evolution of Plant Terpenoid Chemical Diversity

**DOI:** 10.3389/fpls.2019.01166

**Published:** 2019-10-01

**Authors:** Prema S. Karunanithi, Philipp Zerbe

**Affiliations:** Department of Plant Biology, University of California Davis, Davis, CA, United States

**Keywords:** terpenoids, terpene synthases, plant specialized metabolism, plant chemical diversity, terpenoid biosynthesis, natural products

## Abstract

Terpenoids comprise tens of thousands of small molecule natural products that are widely distributed across all domains of life. Plants produce by far the largest array of terpenoids with various roles in development and chemical ecology. Driven by selective pressure to adapt to their specific ecological niche, individual species form only a fraction of the myriad plant terpenoids, typically representing unique metabolite blends. Terpene synthase (TPS) enzymes are the gatekeepers in generating terpenoid diversity by catalyzing complex carbocation-driven cyclization, rearrangement, and elimination reactions that enable the transformation of a few acyclic prenyl diphosphate substrates into a vast chemical library of hydrocarbon and, for a few enzymes, oxygenated terpene scaffolds. The seven currently defined clades (a-h) forming the plant TPS family evolved from ancestral triterpene synthase- and prenyl transferase–type enzymes through repeated events of gene duplication and subsequent loss, gain, or fusion of protein domains and further functional diversification. Lineage-specific expansion of these TPS clades led to variable family sizes that may range from a single TPS gene to families of more than 100 members that may further function as part of modular metabolic networks to maximize the number of possible products. Accompanying gene family expansion, the TPS family shows a profound functional plasticity, where minor active site alterations can dramatically impact product outcome, thus enabling the emergence of new functions with minimal investment in evolving new enzymes. This article reviews current knowledge on the functional diversity and molecular evolution of the plant TPS family that underlies the chemical diversity of bioactive terpenoids across the plant kingdom.

## Introduction

Among the wealth of small molecule natural products, terpenoids (also referred to as isoprenoids) form an especially diversified and evolutionary ancient superfamily, which likely emerged alongside the formation of primitive membranes at the very origins of cellular life (Ourisson and Nakatani, 1994). Ubiquitous presence of terpenoids in membranes supports this hypothesis and suggests that ancient archaebacterial diphytanylglycerol ether membrane components, polyprenols, and derived steranes and sterols represent early terpenoid predecessors (Ourisson and Nakatani, 1994; Rohmer and Bisseret, 1994; Van De Vossenberg et al., 1998; Matsumi et al., 2011). From this origin, the staggering diversity of the terpenome has arisen, comprising more than 80,000 compounds (Christianson, 2018) that are widespread across living organisms, including archaea (Matsumi et al., 2011), bacteria (Yamada et al., 2012), fungi (Schmidt-Dannert, 2015), social amoeba (Chen et al., 2016; Chen et al., 2019), marine organisms (Gross and König, 2006), insects (Beran et al., 2016; Lancaster et al., 2018), and plants (Gershenzon and Dudareva, 2007; Tholl, 2015). Plants are the champions of producing different terpenoid structures (Tholl, 2015). This includes a few isoprenoid derivatives with essential roles in plant growth and development such as gibberellins, brassinosteroids, carotenoids, and chlorophylls (Pallardy, 2008; Tripathy and Pattanayak, 2012). Conversely, the vast majority of plant terpenoids represent specialized metabolites that are dedicated to mediating interorganismal interactions or environmental defense and adaptation (Gershenzon and Dudareva, 2007; Tholl, 2015). For example, many terpenoids exhibit potent toxicity and serve as core components of chemical defenses against herbivores, insect pests, and microbial pathogens (Keeling and Bohlmann, 2006a; Vaughan et al., 2013; Schmelz et al., 2014). In addition, functions in allelopathic interactions and roles in abiotic stress responses have been reported (Lopez et al., 2008; Kato-Noguchi and Peters, 2013; Vaughan et al., 2015). Terpenoid bioactivities in cooperative interactions are equally diverse, including various volatile terpenoids essential for attracting pollinators and seed dispersers, as well as in mediating plant–plant and plant–microbe interactions that impact plant fitness (Dudareva and Pichersky, 2000; Pichersky and Gershenzon, 2002; Heil and Ton, 2008; Agrawal and Heil, 2012). Driven by selective pressures to adapt to the biotic and abiotic environments of the ecological niche occupied by individual plant species, specialized terpenoid metabolism has undergone an expansive evolutionary divergence, resulting in often lineage-specific pathways and products (Chen et al., 2011; Tholl, 2015; Zerbe and Bohlmann, 2015). Biosynthesis and accumulation of these compounds also are typically restricted to only a subset of organs, tissues, or developmental stages and may be tightly regulated by internal or external stimuli, granting plants the ability to fine-tune the deployment of terpenoids for mediating dynamic interactions with the environment (Keeling and Bohlmann, 2006a; Tholl, 2006; Schmelz et al., 2014). Owing to their diverse bioactivities, terpenoid-forming plants and their products have a long history of exploitation for human benefit. Historically, large-scale extraction of terpenoid resins from coniferous trees has been a resource for producing “turpentine”—giving the metabolite class its name—and continue to be of economic relevance for the manufacture of biopolymers and inks (Bohlmann and Keeling, 2008). Other uses of plant terpenoids span various industrial sectors, including flavors and fragrances (Lange et al., 2011; Schalk et al., 2012; Philippe et al., 2014; Celedon and Bohlmann, 2016), pharmaceuticals and cosmetics (Paddon et al., 2013; Pateraki et al., 2017; Booth and Bohlmann, 2019; Zager et al., 2019), biofuels (Peralta-Yahya et al., 2012; D’espaux et al., 2015), and natural rubber (Oh et al., 2000; Cornish and Xie, 2012; Qu et al., 2015).

**Figure 1:**
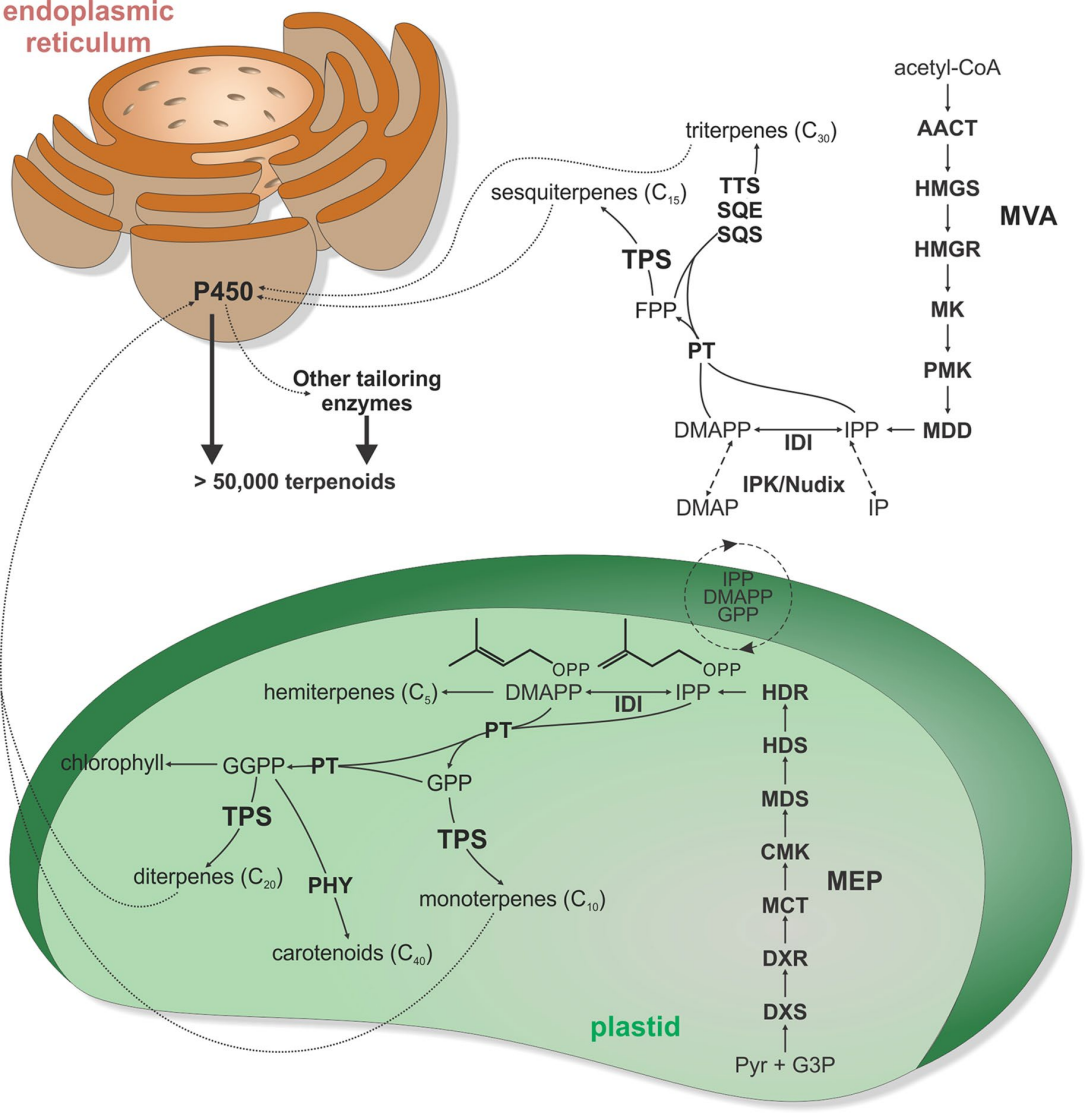
Schematic overview of major terpenoid biosynthetic pathways. All terpenoids are derived from two isomeric 5-carbon precursors, isopentenyl diphosphate (IPP), and dimethylallyl diphosphate (DMAPP). In turn, IPP and DMAPP are formed *via* two pathways, the cytosolic mevalonate (MVA) pathway originating from acetyl-CoA and the pyruvate and glyceraldehyde-3-phosphate (G3P)–derived 2-C-methyl-D-erythritol-4-phosphate (MEP) pathway located in the plastids. However, active transfers of IPP, DMAPP, GPP, and FPP across the plastidial membrane enable some degree of pathway cross-talk. In addition, interconversion of IPP and DMAPP with their respective monophosphate forms IP and DMAP by IP kinase (IPK) and Nudix hydrolase enzymes can impact pathway flux in terpenoid metabolism. Except for isoprene and hemiterpene (C_5_) biosynthesis, condensation of IPP and DMAPP units generates prenyl diphosphate intermediates of different chain length. Condensation of IPP and DMAPP yields geranyl diphosphate (GPP) as the precursor to monoterpenoids (C_10_), fusing GPP with an additional IPP affords the sesquiterpenoid (C_15_) precursor farnesyl diphosphate (FPP), and fusing FPP with IPP generates geranylgeranyl diphosphate (GGPP) en route to diterpenoids (C_20_). Furthermore, condensation of two FPP or two GGPP molecules forms the central substrates of triterpenoid (C_30_) and carotenoids (C_40_), respectively. Terpene synthases (TPS) are key gatekeepers in the biosynthesis of C_10_–C_20_ terpenoids, catalyzing the committed scaffold-forming conversion of the respective prenyl diphosphate substrates into a range of hydrocarbon or oxygenated structures. These TPS products can then undergo various oxygenations through the activity of cytochrome P450 monooxygenases (P450), followed by further possible functional decorations, ultimately giving rise to more than 80,000 distinct natural products. AACT, acetoacetyl-CoA thiolase; CMK, 4-diphosphocytidyl-2-C-methyl-D-erythritol kinase; DXR, 1-deoxy-D-xylulose 5-phosphate reductase; DXS, 1-deoxy-D-xylulose 5-phosphate synthase; HDR, (E)-4-hydroxy-3-methyl-but-2-enyl diphosphate reductase; HDS, (E)-4-hydroxy-3-methyl-but-2-enyl diphosphate synthase; HMGR, 3-hydroxy-3-methylglutaryl-CoA reductase; HMGS, 3-hydroxy-3-methylglutaryl-CoA synthase; IDI, isopentenyl diphosphate isomerase; MCT, MEP cytidyltransferase; MDD, mevalonate-5-diphosphate decarboxylase; MDS, 2-C-methyl-D-erythritol 2,4-cyclodiphosphate synthase; MK, mevalonate kinase; P450, cytochrome P450-dependent monooxygenase; PHY, phytoene synthase; PMK, phosphomevalonate kinase; PT, prenyl transferase; SCS, squalene synthase; SQE, squalene epoxidase; TPS, terpene synthase; TTS, triterpene synthase.

The biological and economic relevance of terpenoids has fostered long-standing efforts in understanding the metabolic enzymes that generate terpenoid chemical diversity. Following common metabolic patterns of scaffold-forming and tailoring reactions in specialized metabolism (Anarat-Cappillino and Sattely, 2014), terpenoid biosynthesis proceeds through conversion of central 5-carbon isoprenoid precursors into a range of core scaffolds that are then functionally elaborated to generate the diversity of terpenoid bioactivities (Davis and Croteau, 2000; Chen et al., 2011; Hamberger and Bak, 2013; Zerbe and Bohlmann, 2015) (**Figure 1**). Functionally distinct enzyme families of terpene synthase (TPS) and cytochrome P450 monooxygenase (P450) enzymes are the major drivers of scaffold formation and functional modifications, respectively (Peters, 2010; Chen et al., 2011; Nelson and Werck-Reichhart, 2011; Zerbe and Bohlmann, 2015; Banerjee and Hamberger, 2018; Bathe and Tissier, 2019) (**Figure 1**). In particular, TPSs serve as the gatekeepers of species-specific terpenoid pathways, catalyzing stereo-specific carbocation cascades that transform a handful of common prenyl diphosphate substrates into the core scaffolds of numerous structurally distinct terpenoid groups. Recent years have witnessed groundbreaking advances in genomics and biochemical tools that have enabled the discovery of TPS and P450 enzymes at an unprecedented scale and can be combined with versatile metabolic engineering approaches toward producing a broader range of terpenoid bioproducts (Keasling, 2012; Kitaoka et al., 2015; Mafu and Zerbe, 2018). Building on comprehensive reviews on terpenoid biological function, regulation, and biochemistry (Dudareva and Pichersky, 2000; Tholl, 2006; Gershenzon and Dudareva, 2007; Hamberger and Bak, 2013; Lange and Turner, 2013; Schmelz et al., 2014; Tholl, 2015), this review focuses on recent advances in the knowledge of terpenoid biosynthesis and the evolutionary divergence of the TPS family.

## Metabolic Origin of Terpenoid Precursors

### Biosynthesis of C_5_ Isoprenoid Building Blocks

The metabolic origin of all terpenoids centers around the assembly of multiples of the common C_5_ isoprenoid precursor isopentenyl diphosphate (IPP) and its double-bond isomer dimethylallyl diphosphate (DMAPP) (Lange et al., 2000; Christianson, 2008). Unlike most microbial organisms, plants utilize two distinct pathways for producing these building blocks: the acetyl-CoA-derived cytosolic mevalonate (MVA) pathway and the pyruvate-derived plastidial 2-*C*-methyl-D-erythritol-4-phosphate (MEP) pathway (McGarvey and Croteau, 1995; Hemmerlin et al., 2012) (**Figure 1**). Presence of the MVA pathway in archaea (albeit with some species being devoid of some pathway enzymes) and phylogenetic relatedness of MVA pathway genes across multiple taxa provide evidence that the MVA pathway represents the ancestral isoprenoid-metabolic route that was present in the last common ancestor and has been vertically transmitted to the descendants (Lombard and Moreira, 2011; Matsumi et al., 2011). By contrast, the plastidial MEP pathway was likely acquired through horizontal gene transfer from different bacterial progenitors such as cyanobacteria and various proteobacteria (Lange et al., 2000). The metabolic expense of retaining two IPP/DMAPP-metabolic pathways in plants holds apparent advantages by enabling broader ability to evolve specialized terpenoid pathways and better control of compartment-specific isoprenoid pools toward MEP-derived mono- and di-terpenoids, carotenoids, plastoquinones and chlorophyll in plastids and MVA-derived sesquiterpenoids, sterols, brassinosteroids, and triterpenoids. This physical separation of downstream pathways has been supported—for example, by genome-wide co-expression studies in *Arabidopsis* that showed minimal interaction between MVA and MEP genes (Wille et al., 2004; Vranova et al., 2013; Rodriguez-Concepcion and Boronat, 2015). In addition, presently known pathway connections appear to be largely negative in nature, where transcriptional activation of MEP genes correlate with the repression of MVA genes and *vice versa* (Ghassemian et al., 2006; Rodriguez-Concepcion and Boronat, 2015). On the other hand, metabolic compensation, for example, of cytosolic sterol biosynthesis through the MEP pathway has been described (Hemmerlin et al., 2003; Laule et al., 2003). Indeed, cross-talk between both pathways *via* exchange of IPP, DMAPP, and C_10–15_ prenyl diphosphate intermediates has been demonstrated in several species (Hemmerlin et al., 2003; Laule et al., 2003; Opitz et al., 2014; Mendoza-Poudereux et al., 2015), indicating that the metabolic fate of MEP- and MVA-derived IPP and DMAPP is not as clear cut. For example, isotope-labeling studies demonstrated incorporation of MEP-derived IPP/DMAPP into both mono- and sesqui-terpenoids in snapdragon (*Antirrhinum majus*) and carrot (*Daucus carota*) (Dudareva et al., 2005; Hampel et al., 2005). Similar work in cotton (*Gossypium hirsutum*) showed contribution of the MVA pathway to C_10_–C_40_ terpenoid biosynthesis (Opitz et al., 2014).

How the cross-membrane exchange of MEP and MVA intermediates is coordinated also requires further investigation. Transport of IPP and GPP across the plastidial membrane has been observed in isolated plastids (Soler et al., 1993; Bick and Lange, 2003; Flugge and Gao, 2005), but transporters or alternate transfer mechanisms are thus far unknown (Pick and Weber, 2014). Recent studies further illustrated that terpenoid biosynthesis *via* the MVA and MEP pathways is not solely routed through IPP and DMAPP but can involve a pool of the respective isopentenyl and dimethylallyl monophosphates, IP, and DMAP (Henry et al., 2015; Henry et al., 2018). The IP and IPP pools are controlled by two enzymes families, IP kinases and Nudix hydrolases, that catalyze the phosphorylation and hydrolysis of IP and IPP, respectively (Henry et al., 2015; Henry et al., 2018) (**Figure 1**). IP kinases were first discovered in archaea and Chloroflexi as an alternate pathway for isoprenoid biosynthesis (Dellas et al., 2013). More recently, IP kinase homologs were shown to be widely distributed in plant genomes, where they occur alongside the complete set of MVA and MEP pathway genes (Vannice et al., 2014). In *Arabidopsis*, IP kinase was shown to localize at the cytosol and regulate the formation of both MVA- and MEP-derived terpenoids as based on reverse genetic studies (Henry et al., 2015). *AtI*PK knockout in *Arabidopsis* using T-DNA insertion lines caused a significant decrease in the levels of sterols (37–50%) and sesquiterpenes produced (25–31%). Conversely, overexpression of *At*IPK in transgenic *Nicotiana tabacum* led to a 3-fold and 2-fold increase in sesquiterpenes and monoterpenes, respectively. Further efforts to understand the formation of IP/DMAP in plants identified a role of Nudix hydrolases, a superfamily of two-domain hydrolases/peptidases broadly found in bacteria, animals, and plants (Bessman et al., 1996; Ogawa et al., 2005; Magnard et al., 2015; Henry et al., 2018). *In vitro* and genetic studies of the two cytosolic Nudix hydrolases in the *Arabidopsis* genome, *At*Nudx1 and *At*Nudx3, demonstrated their efficiency in dephosphorylating IPP and DMAPP (Henry et al., 2018). *Arabidopsis* T-DNA insertion gene knock-down and knock-out lines of *At*Nudx1 and *At*Nudx3 resulted in increased production of sesquiterpenes (28–60%), monoterpenes (148–503%), and sterols (∼50%) whereas overexpression of these enzymes in *N. tabacum* resulted in decreased production of monoterpenes (∼50%) and sesquiterpenes (57–88%). Although understanding the broader relevance of IP kinase and Nudix hydrolase genes in plant terpenoid metabolism requires further studies, these collective findings highlight the potential of these pathway reactions to possibly function as additional regulatory mechanisms for balancing the IP/DMAP and IPP/DMAPP pools in the biosynthesis of terpenoids and other isoprenoids (Henry et al., 2015; Henry et al., 2018). Given the dramatic impact of modulating IP kinase and Nudix hydrolases gene expression on pathway productivity, combined tailoring of these pathway nodes holds promise for advanced terpenoid pathway engineering.

### Biosynthesis of Prenyl Diphosphate Precursors

**Figure 2:**
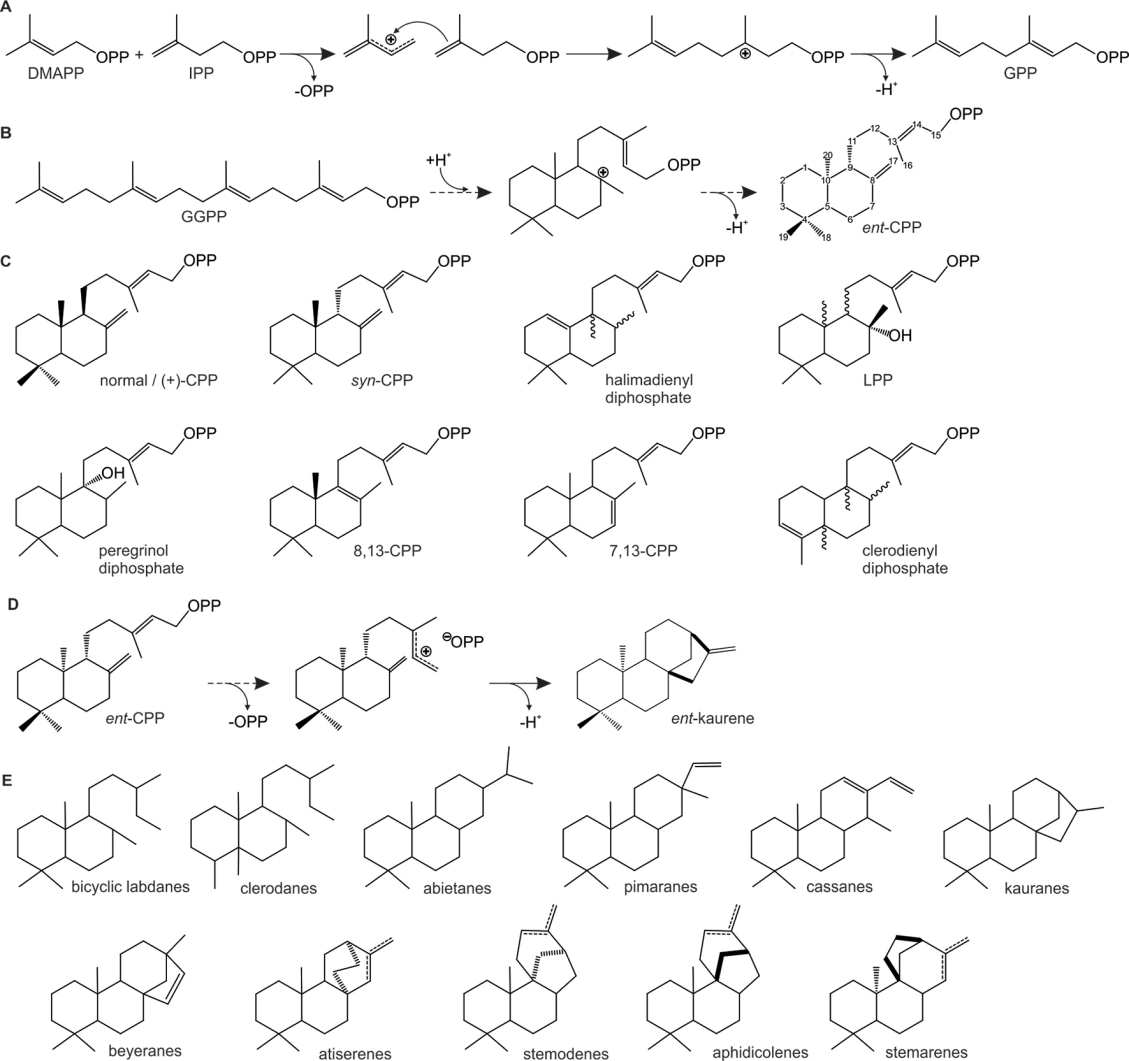
Schematic overview of representative carbocation-driven reactions catalyzed by prenyl transferases (PTs) and diterpene synthases (TPSs). **(A)** PT catalyzed head-to-tail condensation of isopentenyl diphosphate (IPP) with its positional isomer dimethylallyl diphosphate (DMAPP) *via* ionization of the allylic diphosphate ester bond (OPP) and subsequent coupling between the resulting carbocation and the C3–C4 double bond of IPP. Deprotonation of the carbocation intermediate yields geranyl diphosphate (GPP). **(B**–**C)** Conversion of geranylgeranyl GGPP by class II diTPSs using protonation-initiated cyclization of GGPP to facilitate scaffold rearrangement into bicyclic prenyl diphosphates of *ent*-copalyl diphosphate (*ent*-CPP) **(B)** and related scaffolds of distinct stereochemistry and hydroxylation **(C)**. **(D**–**E)** Class I diTPS-catalyzed conversion of bicyclic prenyl diphosphate intermediates *via* ionization of the diphosphate moiety and subsequent cyclization and rearrangement through various 1,2-hydride and methyl migrations to form, for example, *ent*-kaurene **(D)** and a range of other labdane diterpene scaffolds **(E)**.

Downstream of the IPP and DMAPP biosynthesis, prenyl transferases (PTs) catalyze the sequential condensation of isoprenoid units *via* ionization of the allylic diphosphate ester and subsequent rearrangement of the resulting carbocation to generate prenyl diphosphate metabolites of distinct chain length that serve as universal terpenoid precursors (Nagel et al., 2019) (**Figure 1**). Head-to-tail condensation (C4–C1 alkylation) reactions lead to C_10_ (geranyl diphosphate, GPP), C_15_ (farnesyl diphosphate, FPP), and C_20_ (geranylgeranyl diphosphate, GGPP) intermediates as precursors in mono-, sesqui-, and di-terpenoid metabolisms, respectively (**Figure 2A**). Notably, dimerization has been shown to be a major factor impacting PT activity and product specificity. For example, GPP synthases from *Mentha piperita*, *A. majus*, and *Clarkia breweri* require formation of a heterodimer of a small and a large subunits for their enzyme function (Burke et al., 1999; Tholl et al., 2004). Interaction of GPP small subunits with GGPP synthases from *Abies grandis* and *Taxus canadensis* were further shown to modify GGPP synthase product specificity in favor of forming shorter C_10_ chains (Burke and Croteau, 2002). Similarly, interaction of a *cis*-PT with an unusual *cis*-PT-like scaffolding enzyme was shown be a key function in rubber biosynthesis in lettuce (*Lactuca sativa*) (Qu et al., 2015). As alternative routes to the common head-to-tail condensation reactions, C_30_ and C_40_ prenyl diphosphates are formed *via* head-to-head condensation of FPP or GGPP through the activity of squalene synthases or phytoene synthases en route to triterpenoids and carotenoids, respectively (Christianson, 2008). Similarly, catalysis of non-head-to-tail or irregular C1′-2-3 isoprenoid condensation can occur as exemplified by a PT from *Chrysanthemum cinerariaefolium* that forms the irregular monoterpene chrysanthemyl diphosphate (Rivera et al., 2001). Recent years further revealed the biosynthetic enzymes forming plant prenyl diphosphate products of other chain length, including C_25_ intermediates en route to the rare group of defensive sesterterpenoids in *Brassicaceae* and a few other species (Luo et al., 2010; Nagel et al., 2015; Huang et al., 2017; Chen et al., 2019). *Arabidopsis* studies further illustrated a *trans*-type polyprenyl diphosphate synthase with the capacity to produce variable chain length (C_25_–C_45_) products (Hsieh et al., 2011).

In addition to product chain length, PT enzymes can be distinguished based on the *cis*- or *trans*-C–C double-bond configuration of their product (Liang et al., 2002). Although PTs utilize the same isoprenoid substrates, *cis*- and *trans*-type PTs differ in their protein structure and signature motifs that control catalytic specificity (Liang et al., 2002). Similar to class I TPS enzymes (described below), *trans*-PTs feature two aspartate-rich motifs, FARM (DDx_2/4_D) and SARM (DDx_2_D), which are critical for substrate binding, whereas *cis*-PTs lack these motifs, and substrate binding is controlled by Asp and Glu residues broadly distributed within the active site (Liang et al., 2002). Medium- and long-chain (≥C_30_) PTs more commonly produce *cis*-prenyl diphosphate compounds, most prominently represented by *cis*-PTs of rubber biosynthesis (Asawatreratanakul et al., 2003; Cornish and Xie, 2012; Chakrabarty et al., 2015; Qu et al., 2015). In contrast, the majority of short-chain (C_10–25_) prenyl diphosphates occur in the *trans* configuration. However, a number of *cis*-PTs have been identified in certain species that produce intermediates featuring *cis-* and *trans-*configured double bonds. For example, short-chain *cis*-PTs were identified in tomato (*Solanum* sp.) (Sallaud et al., 2009; Schilmiller et al., 2009; Akhtar et al., 2013; Matsuba et al., 2015). In the wild tomato variety *Solanum* *habrochaites*, an FPP synthase, zFPS, was demonstrated to form the *cisoid* FPP form *Z,Z*-FPP, which is further converted by a *Z,Z*-FPP-specific TPS (SBS) to form (+)-α-santalene, (+)-endo-β-bergamotene, and (−)-endo-α-bergamotene (Sallaud et al., 2009). Notably, zFPS localizes to the chloroplast, contrasting the commonly cytosolic localization of *trans*-FPP synthases (Sallaud et al., 2009; Schilmiller et al., 2009; Akhtar et al., 2013). Further gene discovery studies in cultivated tomato (*Solanum lycopersicum*) identified neryl-diphosphate synthase 1 (NDPS1) catalyzing the formation of a *cis*-neryl diphosphate (NPP) as a precursor for a range of monoterpenoids in addition to the canonical *trans*-substrate GPP (Schilmiller et al., 2009; Gutensohn et al., 2013). More recently, a diterpenoid-metabolic cluster was reported in cultivated tomato that included an unusual *cis*-PT (CPT2) that produces the *cisoid* C_20_ GGPP variant *Z,Z,Z*-nerylneryl diphosphate (NNPP) (Matsuba et al., 2015). NNPP conversion by a TPS (TPS21) and a P450 (CYP71BN1) located within this cluster yielded the unusual diterpenoid lycosantalonol (Matsuba et al., 2015). Combinatorial functional analysis of class II TPSs known to convert *transoid* *E*,*E*,*E*-GGPP showed a broad capacity to also convert NNPP (Jia and Peters, 2017; Pelot et al., 2019). Together, the identification of *cis*-PT enzymes in an increasing number of species and the capacity of several TPSs to convert both *transoid* and *cisoid* prenyl diphosphate substrates may suggest that *cis*-prenyl diphosphate–derived terpenoids are more widely distributed than previously assumed.

## Evolution of Terpene Synthases Drives Terpenoid Chemical Diversity

**Figure 3:**
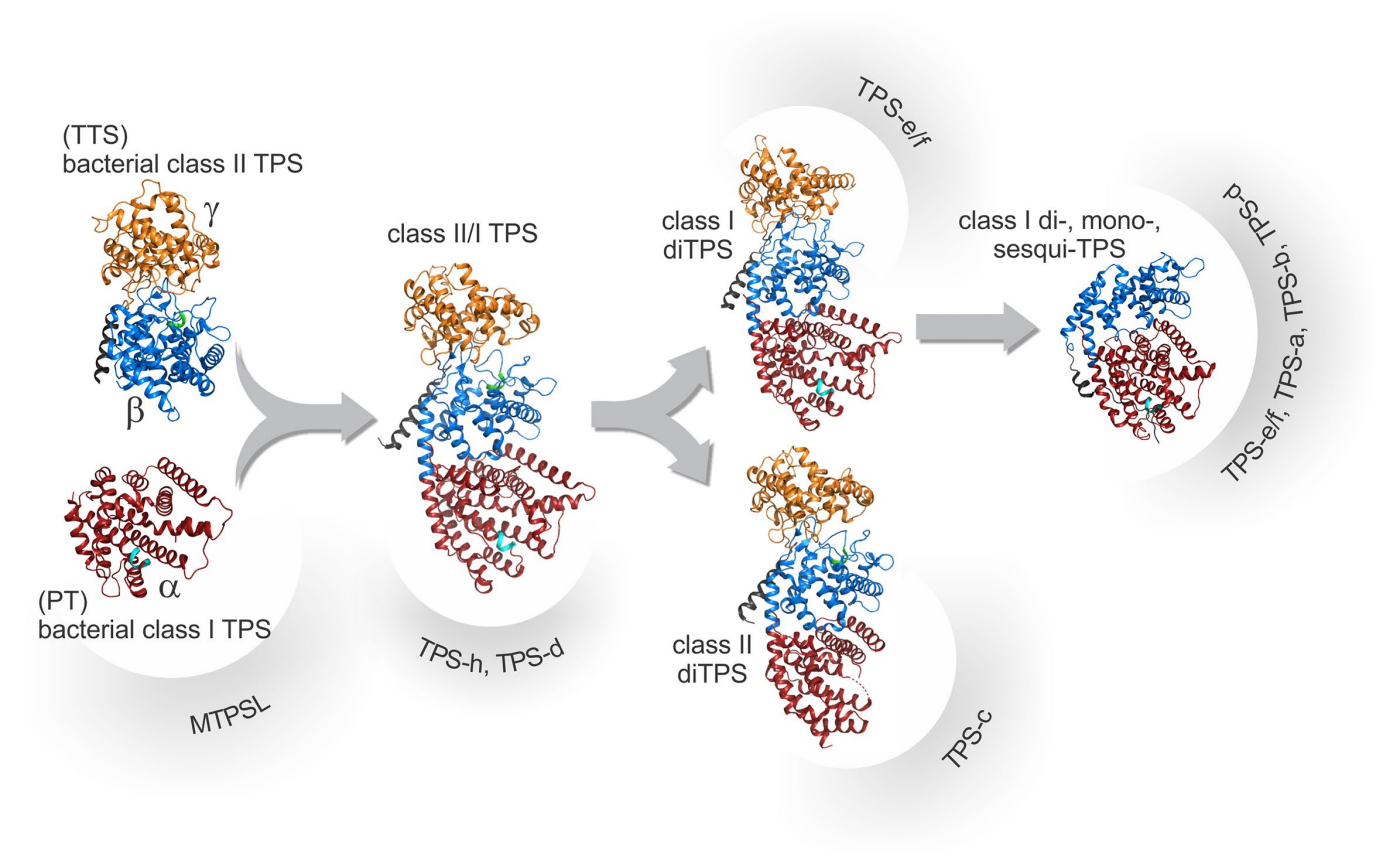
Schematic overview of the proposed structural and evolutionary relationships among terpene synthases (TPSs) as based on known protein structures. Proposed progenitors of TPSs include ancestral bacterial class II diTPSs with a signature α-barrel βγ-domain harboring a catalytic DxDD motif, here exemplified by the *ent*-copalyl diphosphate synthase from *Streptomyces platensis* (PDB 5BP8; Rudolf et al., 2016). These, in turn, are related to ancestral triterpene synthases (TTS) and bacterial class I diterpene synthases (diTPSs) that adopt the signature α-domain structure with a conserved DDx_2_D motif, here exemplified by *ent-*kaurene synthase from *Bradyrhizobium japonicum* (PDB 4W4R; Liu et al., 2014) closely related to ancestral prenyltransferases (PT). Fusion of the ancestral monofunctional genes will have given rise to diTPSs with three helical domains (αβγ) represented here by *Abies grandis* abietadiene synthase (PDB 3S9V; Zhou et al., 2012a). Duplication and subsequent loss of activity in the βγ- and α-domains, respectively, lead to the emergence of monofunctional plant class II, here represented by *Arabidopsis thaliana ent*-copalyl diphosphate synthase, (PDB 4LIX; Köksal et al., 2014) and class I diTPSs, here represented by *Taxus* *brevifolia* taxadiene synthase (PDB 3P5P; Köksal et al., 2011). Through further loss of the γ-domain and various neo-functionalization and specialization events, the large classes of βα-domain class mono- and sesqui-TPSs will have arisen. Domain colors illustrate the γ-domain (orange), the β-domain (blue), the α-domain (red), as well as the conserved DxDD (green) and DDx_2_D (cyan) motifs.

The family of TPS enzymes governs the committed scaffold-forming C–C bonding and hybridization reactions in the biosynthesis of terpenoid chemical diversity from a handful of acyclic and achiral C_5n_ prenyl diphosphate substrates. At the core of TPS product specificity is the intramolecular rearrangement of highly reactive carbocation intermediates. Although with far greater variation, these reactions are mechanistically analogous to those observed in PT enzymes (**Figure 2**), which suggested an evolutionary relationship between both enzyme families (Gao et al., 2012). Structural studies further strengthened this hypothesis by illustrating that these enzymes share a common TPS fold comprised of variations of three conserved helical domains, α, βα, or γβα (**Figure 3**). Differences in the functionality of these domains distinguish two major classes of TPSs: class II TPSs generate the initial carbocation intermediate *via* substrate protonation and catalyze scaffold rearrangements without cleavage of the diphosphate ester bond, whereas class I TPSs utilize ionization of the diphosphate moiety to form the intermediary carbocation (Davis and Croteau, 2000; Peters, 2010) (**Figures 2B, C**). The class II (βγ) domain adopts a characteristic double α-barrel structure that likely evolved from bacterial class II TPSs, which in turn are related to ancestral class II triterpene synthases such as squalene-hopene cyclase (Cao et al., 2010; Gao et al., 2012; Christianson, 2018) (**Figure 3**). Crystallization of the class II *ent*-copalyl diphosphate (CPP) synthase from *Streptomyces platensis* empirically demonstrated this typical α-barrel βγ-domain structure featuring a Dx_4_E motif closely related to the DxDD signature motif critical for the activity of plant class II diTPSs (Rudolf et al., 2016). Class I activity occurs in the α-helical α-domain (**Figure 3**), predecessors of which will have been ancestral bacterial class I PT and TPS enzymes, as exemplified by the crystal structure of the class I diTPS *ent-*kaurene synthase from *Bradyrhizobium japonicum* that illustrates the presence of the characteristic α-domain fold along with the signature catalytic DDxxD of class I TPSs (Liu et al., 2014). Such consecutively acting *ent*-CPP and *ent*-kaurene synthases are indeed broadly distributed in plant-associated bacteria, including symbiotic rhizobia such as α-, β−, and γ-proteobacteria (*Rhizobiales*) and some phytopathogens such as species of *Xanthomonas* and *Erwinia* (Morrone et al., 2009; Nagel and Peters, 2017; Nagel et al., 2018). Ancestral *ent*-CPP and *ent*-kaurene synthases are core enzymes in the formation of bioactive gibberellin (GA) phytohormones, and all so far characterized bacterial *ent*-CPP and *ent*-kaurene synthases function as part of GA-biosynthetic operons, albeit with some end product variation ranging from GA precursors to bioactive GA_4_ (Nagel and Peters, 2017). Based on the wide distribution of GA-biosynthetic gene clusters in bacteria, it has been suggested that plants acquired the ability to form GAs through ancient events of horizontal gene transfer with soil bacteria (Cao et al., 2010; Smanski et al., 2012), thus providing a selective advantage for phytohormone biosynthesis to control growth and development, as well as a genetic reservoir for the evolution of specialized diterpene metabolites as discussed below.

### Distribution of Plant Microbial-Like Terpene Synthases (MTPSLs)

**Figure 4:**
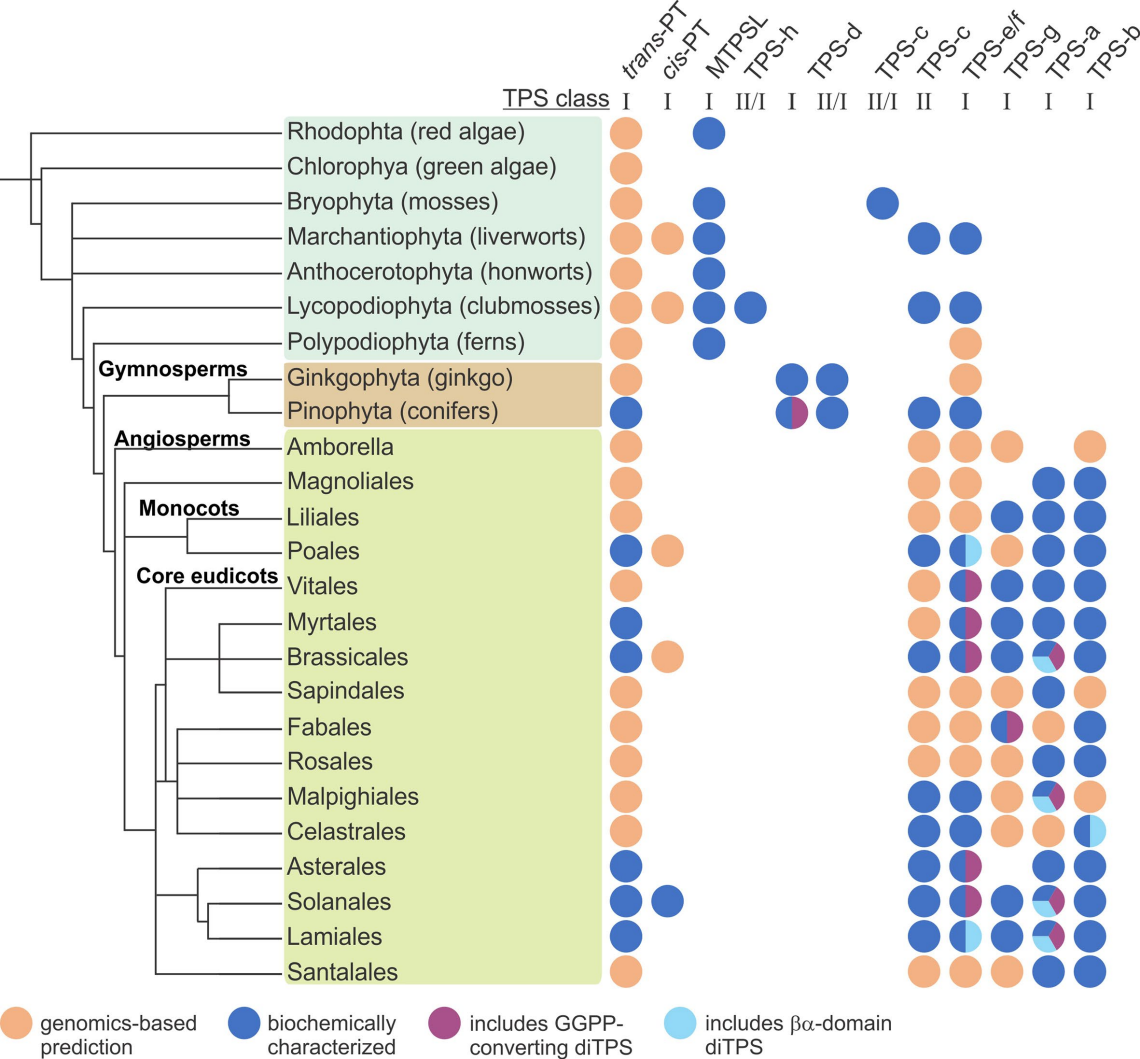
Distribution of terpene-biosynthetic enzyme families across major orders across the plant kingdom. PT, prenyltransferases; MTPSL microbial-type terpene synthases (TPSs); TPS-h, *Selaginella*-specific bifunctional class II/I diterpene synthases (diTPSs); TPS-d, gymnosperm-specific class I mono-, sesqui-, di-TPSs, and bifunctional class II/I diTPSs; TPS-c, monofunctional class II diTPSs; TPS-e/f, monofunctional class I diTPSs; TPS-g, monofunctional class I mono-, sesqui-, and di-TPSs; TPS-a, monofunctional class I sesqui- and di-TPSs; TPS-b, monofunctional class I mono-TPSs.

Recent studies revealed that some plant species retained a previously unknown class of microbial-like TPSs, termed microbial TPS-like (MTPSL). A family of MTPSL was first discovered by Li et al. in the *Selaginella moellendorffii* genome, where they co-occur with classical plant TPSs (Li et al., 2012). The 48 MTPSLs identified in *S. moellendorffii* are phylogenetically more closely related to bacterial and fungal TPS-like sequences and differ from classical plant TPSs on the basis of several key features. Firstly, MTPSLs show a distinct gene structure with a higher variability of introns (0–7) as compared to 12–14 introns in classical plant TPSs (Trapp and Croteau, 2001; Li et al., 2012). Secondly, MTPSLs adopt a single domain α-fold closely resembling the structure of microbial α-domain enzymes rather than βα-domain plant TPSs (Li et al., 2012). Lastly, alongside the common class I DDx_2_D signature motif, MTPSLs contain additional DDx_3_D and DDx_3_ motifs suggesting a distinct evolutionary origin (Li et al., 2012). Despite their structural distinctiveness, *in vitro* enzyme assays demonstrated that MTPSLs form common mono- and sesqui-terpene products, including linalool, germacrene D, and nerolidol that naturally occur in *S. moellendorffii* (Li et al., 2012). Following the discovery of MTPSLs in *S. moellendorffii*, members of this TPS class were also identified in the liverwort *Marchantia polymorpha*, the hornwort *Anthoceros punctatus,* the moss *Sphagnum lescurii*, and the monilophyte ferns *Pityrogramma trifoliata* and *Woodsia scopulina*, suggesting a broader distribution across evolutionary older plant lineages (Jia et al., 2016b; Jia et al., 2018b; Xiong et al., 2018) (**Figure 4**). Functionally active MTPSLs were also identified in the genomes of some red algae (*Rhodophyta*) such as *Laurencia pacifica,* *Porphyridium purpureum*, and *Erythrolobus australicus* (Kersten et al., 2017; Wei et al., 2018). However, expansive genomics studies across several hundred species suggest that MTPSLs are absent in seed plants and green algae (Jia et al., 2016b). Close phylogenetic relationships of MTPSLs with fungal or bacterial TPSs support the evolution of MTPSL genes through multiple events of horizontal gene-transfer events between plants, bacteria, and fungi after the split from the last common ancestor with the green algae lineage (Jia et al., 2016b; Kersten et al., 2017; Jia et al., 2018b; Wei et al., 2018). It can be speculated that the loss of MTPSLs in seed plant lineages is due to the emergence of sesqui-TPS and mono-TPS functions derived from the ancestral bifunctional diTPSs (Jia et al., 2018b).

### Emergence and Diversification of Bifunctional Terpene Synthases

A hallmark event in TPS evolution was the fusion of βγ- and α-domain enzyme classes that gave rise to bifunctional class II/I diTPSs with a βγα-domain architecture (**Figure 3**), providing an apparent evolutionary advantage of improved metabolite channeling of reactive prenyl diphosphate intermediates. Such bifunctional diTPSs have been identified in fungi, mosses, and gymnosperms (Toyomasu et al., 2000; Keeling and Bohlmann, 2006b; Hayashi et al., 2006; Mafu et al., 2011; Zhou et al., 2012a; Fischer et al., 2015; Kumar et al., 2016) but are absent in angiosperms (**Figure 4**). Whether such domain fusions occurred in the bacterial donor or after transfer of monofunctional diTPS genes remains to be resolved. Moreover, the relevant horizontal gene transfer events likely included only a subset of genes rather than entire operons. For example, the bryophyte *Physcomitrella* *patens* contain a single prototypical class II/I diTPS producing *ent*-kaurene and *ent*-hydroxy-kaurene *via* *ent*-CPP as an intermediate (Hayashi et al., 2006). However, *P. patens* lacks additional genes required for producing bioactive GAs and instead forms the GA intermediate *ent*-kaurenoic acid that functions as a growth and developmental regulator (Hayashi et al., 2006). Presence of ancestral bifunctional diTPSs involved in the biosynthesis of GA-related compounds in *P. patens* and related land plants (Hayashi et al., 2006), but not algae (Lohr et al., 2012; Jia et al., 2018b; Wei et al., 2018), places the evolutionary origin of plant diterpene metabolism with the emergence of nonseed plant lineages approximately 450 million years ago. Presumably in the same time period, ancestral *ent*-(OH)-kaurene-producing bifunctional diTPSs underwent neo-functionalization toward the biosynthesis of diterpenoids with specialized functions in a number of species. The moss *Hypnum plumaeforme* contains a bifunctional diTPS that forms *syn*-pimara-7,15-diene, a diterpenoid also present in rice as precursor of anti-microbial and allelopathic momilactones (Wilderman et al., 2004; Kato-Noguchi and Peters, 2013; Schmelz et al., 2014; Okada et al., 2016). Similarly, two class II/I diTPSs have been identified in *S. moellendorffii* that produce miltiradiene *via* the enantiomer of *ent*-CPP (9*R*,10*R*-CPP), namely, normal (9*S*10*S*) CPP or (+)-CPP as an intermediate (*Sm*MDS) and labda-7,13*E*-dien-15-ol (*Sm*CPS/KSL1) *via* labda-15-en-8-ol diphosphate (LPP) (Mafu et al., 2011; Sugai et al., 2011). Although the physiological relevance of these diterpenoids remains elusive, it is plausible that these compounds or derivatives thereof function in disease and pest defense, considering the bioactivity of closely related metabolites in other plant species (Ma et al., 2012; Helmstädter, 2013). In contrast, the role of specialized bifunctional class II/I diTPSs of the gymnosperm-specific TPS-d clade is well established, where these enzymes form the abietane- and pimarane-type labdane scaffolds in the biosynthesis of diterpene resin acids (DRAs) that serve as a durable defense against insect pests and associated fungal pathogens (Keeling and Bohlmann, 2006a; Bohlmann, 2011). Bifunctional abietane– and pimarane-type diTPSs were identified in *Ginkgo biloba* (Schepmann et al., 2001), species of fir (*Abies*) (Peters et al., 2000; Zerbe et al., 2012b), spruce (*Picea*) (Martin et al., 2004), and pine (*Pinus*) (Ro and Bohlmann, 2006; Hall et al., 2013). Notably, all so far identified enzymes utilize the enantiomer of *ent*-CPP (9*R*,10*R*-CPP), namely, normal (9*S*10*S*) CPP or (+)-CPP, as an intermediate en route to the their individual diterpene products. In addition, some species feature diTPSs that have undergone additional neo-functionalization. For example, balsam fir (*Abies balsamea*) contains a *cis*-abienol synthase (AbCAS) that catalyzes conversion of GGPP into *cis*-abienol *via* the C-8 hydroxylated CPP intermediate LPP also observed in *S. moellendorffii* (Mafu et al., 2011; Zerbe et al., 2012b).

The bifunctional diTPSs involved in diterpenoid metabolism of mosses, lycophytes, and gymnosperms are strikingly similar to those identified in fungi, especially in *Ascomycota* and some *Basidiomycota* species where they function in pathogenic or plant growth–promoting pathways (Bomke and Tudzynski, 2009; Quin et al., 2014). A bifunctional diTPS with *ent*-CPP/*ent*-kaurene synthase activity was first identified as a part of a GA-biosynthetic gene cluster in *Gibberella fujikuroi* (genus *Fusarium*), the causal agent of to bakanae disease in rice (*Oryza sativa*) (Tudzynski and Holter, 1998; Toyomasu et al., 2000; Tudzynski, 2005). Beyond GA-biosynthetic diTPSs, certain fungal species also contain enzymes for producing specialized diterpenoids, as exemplified by the bifunctional aphidicolan-16β-ol synthase producing a key precursor to aphidicolin toxins in the pathogenic fungus *Phoma betae* (Toyomasu et al., 2004) or the *ent*-CPP/*ent*-kaurene synthase homologs PaDC1/2 involved in the biosynthesis of phyllocladan-triol in *Phomopsis amygdali* (Toyomasu et al., 2008). Recent studies proposed horizontal gene transfer from a plant to an ancestral *Ascomycota* fungus as the primary mechanism underlying the emergence of fungal class II/I diTPSs (Fischer et al., 2015). This hypothesis is supported by several lines of evidence, including the abundance of mutualistic plant-fungal interactions often involving species containing TPS genes such as *Fusarium*, the presence of diTPS in only some *Ascomycota* and *Basidiomycota* species, and the lack of correlation between the phylogenetic relationships of fungal diTPS and the fungal species containing these genes (Fischer et al., 2015). Interestingly, domain fusions between diterpenoid-biosynthetic enzymes are not limited to bifunctional class II/I diTPSs in fungi and other species but also include other chimeric enzymes that, for example, represent fusions of PT and TPS domains (Minami et al., 2018; Mitsuhashi and Abe, 2018), such as the *P. amygdali* Fusicoccadiene synthase that contains an N-terminal class I TPS domain and a C-terminal PT domain and is involved in the biosynthesis of *Fusicoccin* toxins (Toyomasu et al., 2007).

### Functional Radiation of Monofunctional Terpene Synthases

The absence of bifunctional class II/I diTPSs in angiosperms (Chen et al., 2011) highlights another milestone in the expansion of the TPS family; the duplication and sub-functionalization of ancestral bifunctional γβα-domain diTPSs with one descendent retaining class II (*ent*-CPP synthase) activity in the βα-domain and the other copy acting as a monofunctional class I diTPS (*ent*-kaurene synthase) with a functional α-domain (**Figures 2B, C** and **3**). Early examples of such monofunctional enzymes have been described in *S. moellendorffii*, the liverwort *M. polymorpha* (Li et al., 2012; Kumar et al., 2016), and gymnosperms (Keeling et al., 2010) (**Figure 4**). While ancient vascular plants such as *S. moellendorffii* did not yet use these enzymes for producing bioactive GAs (Aya et al., 2011), monofunctional *ent*-CPP and *ent*-kaurene synthase activities in GA biosynthesis are conserved across vascular plants (Keeling et al., 2010; Chen et al., 2011) (**Figures 2B, D**). In addition to their critical role in phytohormone metabolism, monofunctional *ent*-CPP, and *ent*-kaurene synthases will have served as a major genetic reservoir for the lineage-specific expansion of functionally diverse class II and class I TPSs across the plant kingdom (Zi et al., 2014) (**Figure 4**).

Derived from ancestral *ent*-CPP synthases, the TPS-c clade of class II diTPSs has undergone a relatively moderate diversification with known enzymes differing predominantly in their expression patterns and product-specificity toward a range of alternate stereo- and double-bond isomers and hydroxylated intermediates (Peters, 2010; Zerbe and Bohlmann, 2015) (**Figures 2B, C**). Most prominently, the *ent*-CPP enantiomer (+)-CPP, first identified as an intermediate of gymnosperm class II/I diTPSs (Peters et al., 2000; Martin et al., 2004), is also a core precursor to many labdane-related specialized diterpenoids in various *Lamiaceae* species (Ma et al., 2012; Brückner et al., 2014; Gao et al., 2014; Zerbe et al., 2014; Božić et al., 2015; Cui et al., 2015; Ignea et al., 2016; Scheler et al., 2016) and some *Poaceous* crops such as maize (*Zea mays*) and wheat (*Triticum aestivum*) (Wu et al., 2012; Mafu et al., 2018). Class II diTPSs forming the alternate stereoisomer *syn*-CPP (9*S*,10*R*-CPP) appear to be of narrower taxonomic distribution with current examples limited to some *Poaceous* grasses (Otomo et al., 2004; Xu et al., 2004; Pelot et al., 2018). While *ent*-CPP, (+)-CPP, and *syn*-CPP are the most commonly occurring labdane diterpene precursors, variable series of 1,2-methyl and/or hydride migrations prior to carbocation neutralization can result in other isomeric structures (Peters, 2010). Examples include clerodienyl diphosphate synthases identified in phylogenetically distant plants such as the *Lamiaceae* *Salvia divinorum*, the *Poaceous* grass switchgrass (*Panicum virgatum*), and *Celastraceae* *Tripterygium wilfordii* (Pelot et al., 2016; Chen et al., 2017; Hansen et al., 2017a; Pelot et al., 2018); 7,13-CPP synthases described in *S. moellendorffii* and the *Asteraceae* *Grindelia robusta* (Mafu et al., 2011; Zerbe et al., 2015); and most recently, 8,13-CPP synthases in maize and switchgrass (Murphy et al., 2018; Pelot et al., 2018). In addition to variations in the scaffold rearrangement, a few class II diTPSs evolved the ability to terminate the carbocation *via* oxygenation rather than deprotonation, a function already present in the *A. balsamea* *cis*-abienol synthase that forms LPP as an intermediate (Zerbe et al., 2012b). Here, oxygenation commonly occurs at the C-8 position to yield LPP with several such enzymes known (Falara et al., 2010; Caniard et al., 2012; Zerbe et al., 2013; Pelot et al., 2017). But also a C-9-oxygenated product has been observed that is formed likely *via* alternate 1,2-hydride shift between C-8 and the neighboring methine group of the labda-13-en-8-yl diphosphate carbocation prior to water quenching (Zerbe et al., 2014; Heskes et al., 2018).

While the functional diversification of class II diTPSs has been largely limited to variations in product specificity, the family of class I diTPSs has seen a vast expansion and functional radiation through which the large clades of class I diTPS (gymnosperm TPS-d, angiosperms TPS-e/f), and ultimately, sesqui-TPSs (gymnosperm TPS-d, angiosperm TPS-a) and mono-TPSs (gymnosperm TPS-d, angiosperm TPS-b) have arisen (Chen et al., 2011; Gao et al., 2012) (**Figure 3**). In angiosperms, the predominant blueprint for this class I TPS expansion will have been the repeated duplication and functionalization of ancestral monofunctional *ent*-kaurene synthases within the TPS-e/f clade (Peters, 2010; Zi et al., 2014; Zerbe and Bohlmann, 2015). Beyond the divergence of enzyme products, many specialized class I diTPSs exhibit broad promiscuity for converting different class II diTPS intermediates into the large class of labdane-related diterpenoids (**Figure 2E**). This biochemical capacity enables modular pathway networks where functionally distinct enzymes can act in different combinations to generate a wider spectrum of possible products. It then appears that dividing class II and class I activities into two monofunctional enzymes has provided an evolutionary advantage over the improved intermediate channeling in bifunctional diTPSs that emerged through the ancestral domain fusion events. Numerous examples of species-specific modular diterpenoid-metabolic networks have been described, including the biosynthesis of stress defensive diterpenoid networks in several *Poaceous* crops such as wheat, rice, and maize (Xu et al., 2007a; Morrone et al., 2011; Zhou et al., 2012b; Zerbe et al., 2014; Cui et al., 2015; Fu et al., 2016; Mafu et al., 2018), as well as specialized diterpenoid metabolism in species of *Salvia* and other *Lamiaceae* (Brückner et al., 2014; Zerbe et al., 2014; Cui et al., 2015; Heskes et al., 2018). Further studies on how interconnected pathway branches are regulated will be essential to better understand how metabolic flux is coordinated between general and specialized pathways, as well as specialized pathway branches sharing key intermediates. Unlike angiosperms where such modular pathways of pairwise-acting monofunctional class II and class I diTPSs are the major metabolic strategy (Peters, 2010; Morrone et al., 2011; Mafu et al., 2015; Zerbe and Bohlmann, 2015), specialized diterpenoid metabolism in gymnosperms largely relies on ancestral bifunctional enzymes. However, the existence of modular pathways also in conifers was suggested by the discovery of monofunctional class I diTPSs in jack pine (*Pinus banksiana*) and lodgepole pine (*Pinus contorta*) that derived from bifunctional progenitors and are capable of utilizing the (+)-CPP intermediate produced by bifunctional class II/I enzymes to form a set of pimarane-type labdanes (Hall et al., 2013). Likewise, two groups of predictably monofunctional class I diTPSs that likely evolved from both TPS-d and TPS-e/f diTPSs were recently identified in Western red cedar (*Thuja plicata*) (Shalev et al., 2018). Biochemical analysis of these enzymes may shed light on the distribution of modular class II-class I diTPS reactions in gymnosperm specialized diterpenoid metabolism.

This expansive diversification of class I diTPSs resulted in a multitude of enzymes with altered substrate/product specificity and/or gene expression patterns as a critical contribution to the emergence of species-specific specialized functions (Peters, 2010; Zerbe and Bohlmann, 2015). Mostly, these catalytic alterations resulted in variations of the carbocation rearrangement to yield different pimarane, abietane, clerodane, kaurane, dolabradane, and related labdane scaffolds (**Figure 2E**). While the majority of these diTPS products are hydrocarbon scaffolds, a few class I diTPSs were discovered in recent years that terminate the intermediary carbocation not by deprotonation but *via* hydroxylation in a manner analogous to class II diTPSs that produce C-8 or C-9 hydroxylated prenyl diphosphate products (Peters, 2010; Zerbe and Bohlmann, 2015). Although examples are presently rare, such enzymes seem to be widely distributed across different plant families, including *P. patens* class I/II 16α-hydroxy-kaurane synthase (Hayashi et al., 2006), *S. moellendorffii* labda-7,13*E*-dien-15-ol synthase (Mafu et al., 2011), 13-hydroxy-8(14)-abietene synthases in several gymnosperm species (Keeling et al., 2011), 16α-hydroxy-*ent*-kaurane synthases from *T. wilfordii* (Hansen et al., 2017a) and *Populus trichocarpa* (Irmisch et al., 2015), nezukol synthase from *Isodon rubescens* (Pelot et al., 2017), and *Salvia sclarea* sclareol synthase (Caniard et al., 2012). The regio-specific hydroxylation reactions catalyzed by these diTPSs suggests that the ability of TPSs to ligate a water molecule or hydroxyl group in the nonpolar active site for coordinated carbocation quenching emerged multiple times independently in terpenoid evolution. Notably, sclareol synthase belongs to a recently discovered group of βα-bi-domain class I diTPSs that have undergone loss of the γ-domain (**Figure 3**). Members of this diTPS group form a separate branch in the TPS-e/f clade and have so far been identified in *Poaceae* and *Lamiaceae* species (**Figure 4**), where they almost invariably form specialized terpenoid products and exhibit broad substrate promiscuity, in some cases, such as wheat (*T. aestivum*) KSL5 and maize TPS1 spanning mono-, sesqui-, and di-terpenoid products (Hillwig et al., 2011; Caniard et al., 2012; Zerbe et al., 2013; Zerbe et al., 2014; Fu et al., 2016; Jia et al., 2016a; Pelot et al., 2016; Pelot et al., 2017; Pelot et al., 2018). Close phylogenetic relationships to TPS-e/f diTPSs combined with partial *ent*-kaurene synthase activity of a few enzymes suggest that these βα-domain diTPSs derived more recently from γβα-domain class I enzymes. These βα-bi-domain diTPSs resemble the likely progenitors of not only specialized class I diTPSs but the vast classes of modern βα-domain mono- and sesqui-TPSs. Here, loss of the γ-domain will have been accompanied by various active site modifications toward converting the shorter C_10_ and C_15_ substrates and catalyzing manifold distinct scaffold rearrangements and, in case of the sesqui-TPS family, alteration of enzyme subcellular localization through loss of the N-terminal plastidial transit peptide. Unlike the typically mid-sized diTPS families of 2–30 members, mono- and sesqui-TPS families have undergone a far greater expansion that resulted in diverse families of—for example, 69 TPSs in grape (*Vitis vinifera*) (Martin et al., 2010) and 113 TPS genes in *Eucalyptus* (Külheim et al., 2015), enabling these species to produce an astounding variety of smaller and more volatile terpenoids.

**Figure 5:**
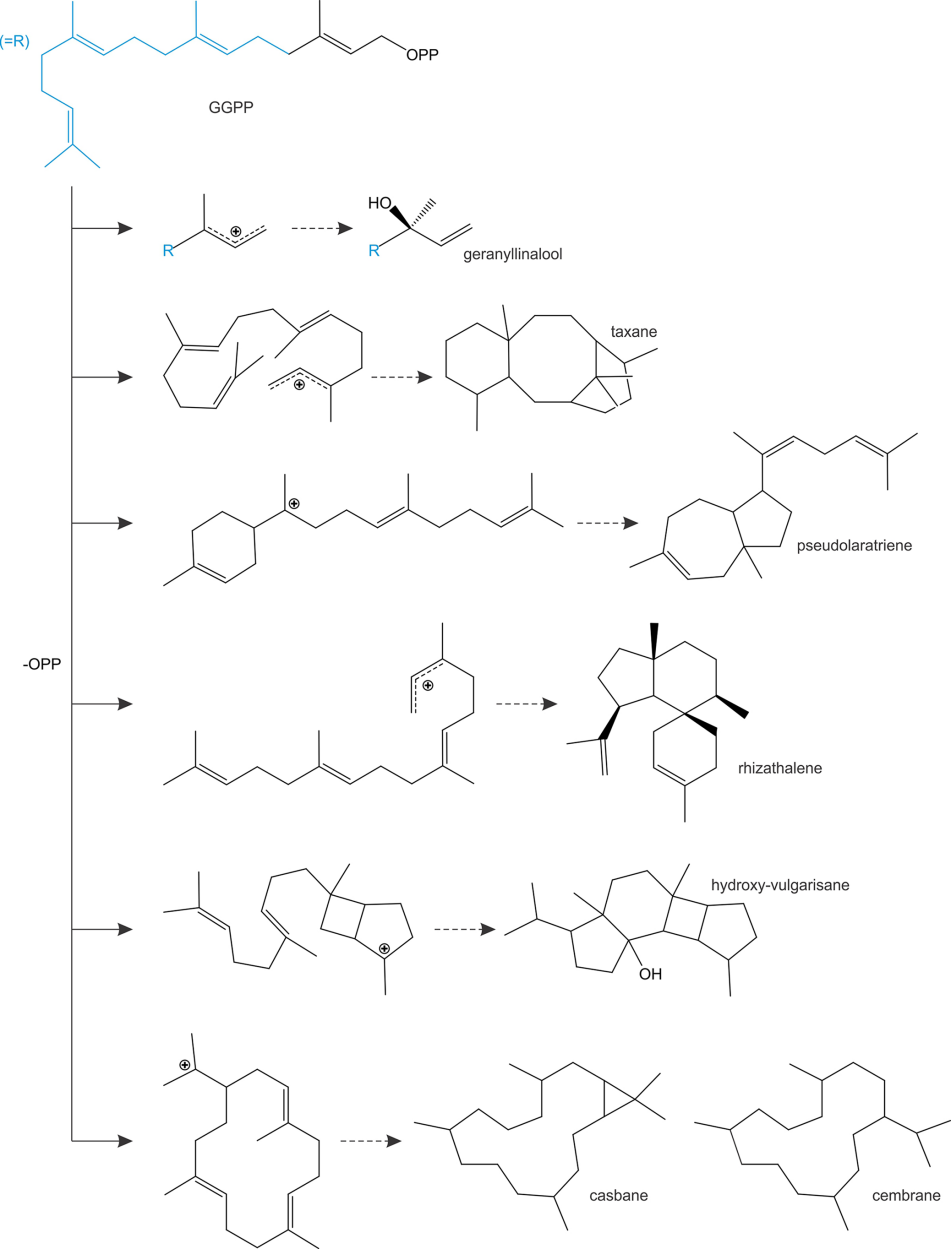
Schematic overview of prominent linear, polycyclic and macrocyclic diterpene scaffolds that are formed through the activity of monofunctional class I diTPSs that are capable of directly converting geranylgeranyl diphosphate (GGPP) as a substrate.

In addition to diTPS converting class II enzyme products, class I diTPSs with the ability to directly convert GGPP as a substrate emerged multiple times during terpenoid evolution and are abundant in a few plant families (**Figure 4**). In gymnosperms, known GGPP-converting monofunctional class I diTPSs are currently limited to taxadiene synthases in species of yew (*Taxus* spp.) that produce the taxane scaffold in the biosynthesis of the chemotherapeutic agent Taxol (Williams et al., 2000) and pseudolaratriene synthase from golden larch (*Pseudolarix amabilis*) that forms an unusual 5–7-ring scaffold en route to the bioactive anti-cancer compound pseudolaric acid B (Mafu et al., 2017) (**Figure 5**). These enzymes adopt the characteristic γβα-domain architecture of gymnosperm diTPSs and phylogenetic analyses clearly indicate that they represent descendants of bifunctional class II/I diTPSs rather than mono- or sesqui-TPSs of the TPS-d clade. In angiosperms, the evolutionary path leading to GGPP-converting diTPSs appears to be more diverse and resulted in enzymes that can produce linear, unusual polycyclic, and macrocyclic scaffolds (Kirby et al., 2010; Vaughan et al., 2013; Falara et al., 2014; King et al., 2014; Luo et al., 2016) (**Figure 5**).

Among these class I diTPSs, geranyllinalool synthases (GLSs) are γβα-domain diTPSs that uniquely catalyze the ionization of the GGPP diphosphate ester bond without subsequent cyclization to occur (**Figure 5**), resulting in an acyclic geranyllinalool intermediate in the biosynthesis of the homoterpenoid (*E*,*E*)-4,8,12-trimethyltrideca-1,3,7,11-tetraene (TMTT) with anti-herbivory activity (Falara et al., 2014). Intriguingly, most of the known GLSs form an ancient branch within the TPS-e/f clade of kaurene(-like) synthases, whereas enzymes from *Fabaceae* species fall into the more divergent TPS-g clade mostly comprised of unusual mono- and sesqui-TPSs (Chen et al., 2011; Falara et al., 2014). Although currently known GLSs are restricted to angiosperm species, phylogenetic studies suggest that TPS-e/f GLSs represent an ancient diTPS family that arose from a common ancestor predating the split of the gymnosperm and angiosperm lineages (Falara et al., 2014). Conversely, *Fabaceae*-specific GLSs of the TPS-g family are likely the result of more recent independent evolutionary events. Alike GLSs, class I enzymes converting GGPP into non-labdane poly- or macro-cyclic scaffolds have been identified in a few angiosperm families (**Figure 4**), including casbene synthases and related macrocyclases in *Euphorbiaceae* (Mau and West, 1994; Kirby et al., 2010; King et al., 2014; Luo et al., 2016), cembratrienol synthases in *Solanaceae* species (Ennajdaoui et al., 2010), *Arabidopsis* rhizathalene synthase (Vaughan et al., 2013), and most recently, 11-hydroxy vulgarisane synthase from the *Lamiaceae* *Prunella vulgaris* (Johnson et al., 2019b) (**Figure 5**). All known members of this group represent βα-bi-domain class I diTPSs and do not belong to the broad TPS-e/f family of class I diTPSs but instead form a distinct branch in the TPS-a clade of sesqui-TPSs (Kirby et al., 2010; King et al., 2014; Johnson et al., 2019b). Two possible evolutionary routes toward these more unusual diTPSs can be envisioned: evolution from TPS-e/f type diTPSs through neo-functionalization and loss of the plastidial signaling peptide or divergence from sesqui-TPS progenitors, which would have involved the re-acquisition of a transit peptide that was previously lost in the evolution of the cytosolic sesqui-TPS family. The latter hypothesis is supported by the close phylogenetic relationship of TPS-a diTPSs and sesqui-TPSs of the TPS-a clade (King et al., 2014; Luo et al., 2016; Johnson et al., 2019b). Such re-evolution events toward diterpenoid-producing TPSs appear not be to restricted to the TPS-a clade, since a monofunctional class I diTPS–producing miltiradiene was identified in *T. wilfordii* that clusters with the TPS-b clade of mono-TPSs rather than with other miltiradiene synthases of the TPS-e/f clade (Hansen et al., 2017a). These collective findings support a highly branched rather than linear evolutionary diversification of TPS functions with many such bifurcations still unknown.

## Catalytic Plasticity of Plant Terpene Synthases

The rapid evolutionary divergence of terpenoid metabolism is aided by the extensive functional plasticity of TPSs. Hence, there has been a long-standing interest in deciphering the mechanisms underlying TPS catalysis and substrate/product specificity. Although TPS structural studies have provided important insight into TPS catalysis, successful crystallization of plant TPSs currently remains limited to a handful of examples, including *Arabidopsis* *ent*-CPP synthase (class II diTPS) (Köksal et al., 2014), *Taxus brevifolia* taxadiene synthase (class I diTPS) (Köksal et al., 2011), *A. grandis* abietadiene synthase (class II/I diTPS) (Zhou et al., 2012a), and a few mono- and sesqui-TPS enzymes (Starks, 1997; Whittington et al., 2002; Hyatt et al., 2007; Shishova et al., 2008; Gennadios et al., 2009; McAndrew et al., 2011). The conserved nature of the TPS fold further has enabled numerous homology-based structure-function studies that provided a deeper insight into the relative ease of functional change in TPS enzymes, where as little as a single residue mutation can alter the active site contour that largely determines product specificity (for detailed reviews see also Gao et al., 2012; Christianson, 2017).

**Figure 6:**
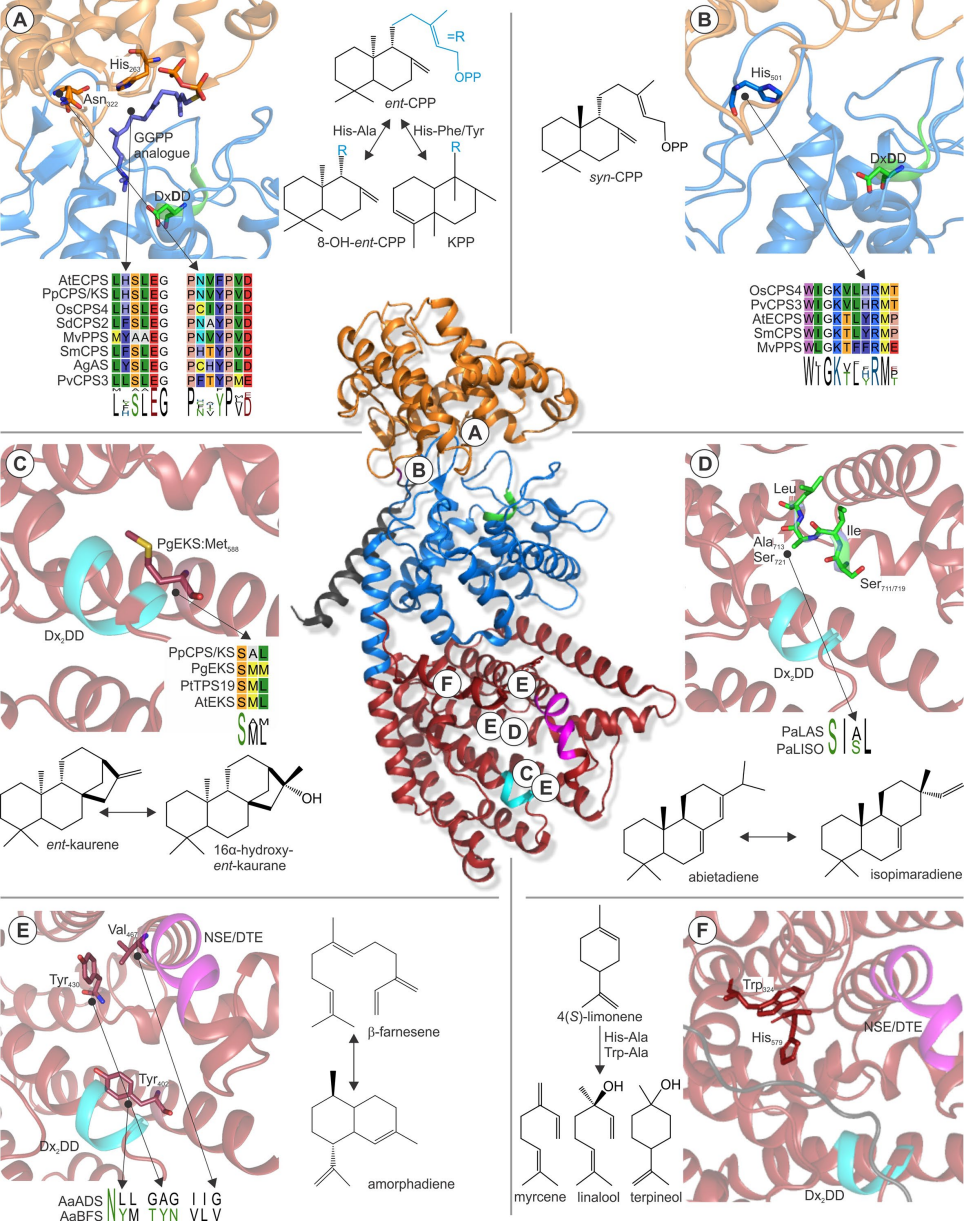
Examples of active site residues with impact on class II or class I TPS product specificity. (**Center**) Structure of *Abies grandis* abietadiene synthase (PDB 3S9V; Zhou et al., 2012a) that adopts the prototypical α-helical TPS fold with variations in three domains γ (orange), β (blue), and α (red). Relative locations of the highlighted active site residues are indicated **A–F**. **(A)** A widely conserved His-Asn dyad is critical for stereo-specificity of *Arabidopsis* *ent*-CPP synthase (PDB 4LIX; Köksal et al., 2014) and other *ent*-CPP synthases (Mann et al., 2010; Potter et al., 2014; Mafu et al., 2015). Substitution of this dyad can result in the formation of the alternate clerodienyl diphosphate product (Potter et al., 2014; Pelot et al., 2016). **(B)** His501 in the rice *syn*-CPP synthase OsCPS4 is critical for the stereo-specific formation of *syn*-CPP and is conserved in known *syn*-CPP synthases but not class II diTPS producing alternate CPP stereoisomers (Potter et al., 2016a; Pelot et al., 2018). **(C)** A conserved Met residue in *ent*-kaurene synthases from *Picea glauca* and other species was shown to control *ent*-kaurene formation (Xu et al., 2007b; Zerbe et al., 2012a). **(D)** Mutational studies of corresponding Ser-Ile-Ala-Leu and Ser-Ile-Ser-Leu motifs located at the hinge region of helix G1/2 of *P. abies* levopimaradiene/abietadiene and isopimaradiene synthase showed their critical role in producing abietane or pimarane scaffolds (Keeling et al., 2008). **(E)** Three residues were identified in the active site of *Artemisia annua* β-farnesene synthase, reciprocal exchange of which to corresponding residues in *A. annua* amorphadiene synthase that control activation (Tyr402), reversion (Tyr430), and restoration (Val476) of cyclization capacity (Salmon et al., 2015). **(F)** Two residues, Trp324 and His579, were shown in limonene synthase of *Mentha spicata* to control the reactions cascade toward the natural product 4(*S*)-limonene (Srividya et al., 2015). The signature catalytic motifs of the class II (DxDD, green) and class I (DDx_2_D, cyan; NSE/DTE, magenta) active sites are highlighted. Protein abbreviations: AtECPS, *Arabidopsis thaliana ent*-copalyl diphosphate (CPP) synthase; OsCPS4, *Oryza sativa syn*-CPP synthase; PvCPS3, *Panicum virgatum* 8,13-CPP synthase; SmCPS, *Salvia miltiorrhiza* (+)-CPP synthase; MvCPS1, *Marrubium vulgare* peregrinol diphosphate synthase; SdCPS2, *Salvia divinorum* clerodienyl diphosphate (KPP) synthase; PpCPS/KS, *Physcomitrella patens* CPP/*ent*-kaurene synthase; AgAS, *Abies grandis* abietadiene synthase; PgEKS, *Picea glauca ent*-kaurene synthase; PtTPS19, *Populus trichocarpa ent*-kaurene synthase; PaLAS, *Picea abies* levopimaradiene/abietadiene synthase; PaISO, *P. abies* isopimaradiene synthase; AaBFS, *Artemisia annua* β-farnesene synthase; AaADS, *A. annua* amorphadiene synthase.

Early work on the class II/I abietadiene synthase from *A. grandis* demonstrated that class II catalysis uses a general aid-base mechanism to bring about the cyclo-isomerization of GGPP, whereby the acid function is provided by the middle aspartate of the conserved DxDD motif (Peters and Croteau, 2002; Prisic et al., 2007; Peters, 2010). Molecular-level insight into this mechanism was recently gained through solving of the crystal structure of *Arabidopsis* *ent*-CPP synthase at high resolution (Köksal et al., 2014). In this study, Köksal and coworkers elegantly demonstrated that proton transfer with this conserved aspartate is enabled by hydrogen-bonded proton wires that link the active site to the bulk solvent (Köksal et al., 2014). Accompanying mutagenesis studies identified the relevant catalytic base in *Arabidopsis* *ent*-CPP synthase as a water molecule that, in turn, is coordinated by a dyad of two histidine and asparagine residues conserved in *ent*-CPP synthases (Mann et al., 2010; Potter et al., 2014) (**Figure 6A**). Alanine substitution of these residues resulted in water quenching rather than deprotonation of the labd-enyl carbocation intermediate to yield hydroxylated *ent*-8-hydroxy-CPP products (Potter et al., 2014; Mafu et al., 2015). The widely conserved relevance of this catalytic dyad has been further supported by site-directed mutagenesis of analogous residues in the bifunctional *ent*-CPP/*ent*-kaurene synthase from *P. patens* and the fungus *Fusarium fujikuroi*, which both resulted in the formation of *ent*-LPP as the class II product (Kawaide et al., 2011; Mafu et al., 2015). Likewise, in the class II/I diTPS, abietadiene synthase from *A. grandis* substitution of a tyrosine corresponding to the catalytic histidine and a nearby histidine led to the redirection of class II specificity from (+)-CPP to 8α-hydroxy-CPP (Criswell et al., 2012). In addition to its critical role in *ent*-CPP synthase activity, residues in the position of this dyad have been shown to impact product specificity in several class II diTPSs. For example, substitution of the *Arabidopsis* *ent*-CPP synthase histidine with phenylalanine or tyrosine rather than Alanine redirected product outcome toward the formation of clerodienyl diphosphate (Potter et al., 2016b) (**Figure 6A**). Strikingly, reciprocal mutagenesis of the corresponding active site positions in recently discovered clerodienyl diphosphate synthases from *S. divinorum* and *T. wilfordii* blocked the series of migration reactions required for forming the clerodane scaffold and resulted in premature deprotonation to form *ent*-CPP (Pelot et al., 2016; Hansen et al., 2017b). Further supporting this evidence, a recent study by Schulte et al. showed that the catalytic dyad in a conserved clade of *Lamiaceae* (+)-CPP synthases is represented by a hydrogen-bonded histidine-tyrosine pair (Schulte et al., 2018). Mutagenesis especially of the tyrosine position in *Salvia miltiorrhiza* (+)-CPP synthase as well as other *Lamiaceae* class II diTPSs showed a dramatic impact on product outcome. Likewise, alanine substitution of a corresponding phenylalanine residue in a 8,13-CPP synthase from switchgrass (PvCPS3) resulted in both positional isomers and hydroxylated forms of the native 8,13-CPP product (Pelot et al., 2018), thus highlighting the relevance of these active site positions in specialized class II diTPSs across diverse plant species.

Another key position contributing to product specificity in class II diTPSs was identified as a histidine (His501) residue in the rice *syn*-CPP synthase OsCPS4, where mutagenesis to aspartate or phenylalanine resulted in additional scaffold rearrangements to form *syn*-halimadienyl diphosphate (Potter et al., 2016a) (**Figure 6B**). A later study on the product specificity of the functionally unique peregrinol diphosphate synthase from *Marrubium vulgare* (MvCPS1) showed that substitution of the corresponding phenylalanine residue and a proximal tryptophan in MvCPS1 also redirected product outcome to yield a halimadienyl diphosphate scaffold (Mafu et al., 2016). These structure-function studies in conjunction with the conservation of the relevant histidine residue in known *syn*-CPP synthases, but not functionally distinct class II diTPSs (**Figure 6B**), support the relevance of this position for controlling biosynthesis of the *syn*-CPP stereoisomer (Potter et al., 2016a). Given the relatively smaller functional range of plant class II diTPSs, knowledge of active site determinants controlling product specificity can facilitate sequence-based prediction of class II diTPS functions as additional residues and functionally distinct enzymes are identified.

By comparison to class II diTPSs, functional annotation of class I TPSs is inherently more complex, due to the larger size and functional diversity of the class I TPS family spanning diterpenoid as well as mono- and sesqui-terpenoid-producing enzymes. However, numerous structure-guided functional studies have provided a deeper understanding of active site determinants that control the fate of intermediary carbocations derived from ionization of the respective linear or bicyclic prenyl diphosphate substrates. Mutational studies of the *ent*-CPP/*ent*-kaurene synthases from *P. patens* and the liverwort *Jungermannia subulata* that produce *ent*-kaurene and 16α-hydroxy-*ent*-kaurane identified an alanine residue that, when substituted for methionine or phenylalanine, blocked formation of 16α-hydroxy-*ent*-kaurane in favor of *ent*-kaurene (Kawaide et al., 2011). Similar studies on *ent*-kaurene synthases from spruce (*Picea glauca*) and polar (*P. trichocarpa*) showed that mutagenesis of the corresponding methionine residues in these enzymes had the reciprocal effect by redirecting product specificity toward 16α-hydroxy-*ent*-kaurane instead of *ent*-kaurene (Zerbe et al., 2012a; Irmisch et al., 2015) (**Figure 6C**), thus suggesting a possible contribution of mutations at this position to the evolution of dedicated *ent*-kaurene synthases.

Another key active site segment that impacts class I TPS product specificity is a small hinge region between helix G1/2 (**Figure 6D**). This helix break is already present in ancestral squalene synthases, and recent structural studies of a bacterial hedycaryol sesqui-TPSs illustrated a role of this helix break in generating a negative electrostatic potential that contributes to carbocation stabilization during catalysis (Pandit et al., 2000; Baer et al., 2014). For example, mutational analysis of a pair of paralogous class II/I diTPSs from Norway spruce (*Picea abies*) illustrated that reciprocal exchange of a largely conserved SIAL/SISL motif located at this hinge region resulted in the complete interconversion of the respective abietadiene and isopimaradiene synthase activities (**Figure 6D**) (Keeling et al., 2010). A similar scenario was observed in several *ent*-kaurene synthases, where mutagenesis of a conserved isoleucine residue at this helix break mitigates formation of a tetracyclic kaurane structure and instead yielded tricyclic *ent*-pimaradiene scaffolds as demonstrated in enzymes from rice, *Arabidopsis*, spruce, and *P. patens* (Wilderman and Peters, 2007; Xu et al., 2007b; Zerbe et al., 2012a). Reciprocal mutagenesis of a corresponding threonine residue in the specialized rice class I diTPS OsKSL5 shifted catalysis from forming *ent*-pimaradiene to producing *ent*-isokaurene and other tetracyclic scaffolds, further supporting the role of residues in this position in controlling the fate of the intermediary *ent*-pimarenyl carbocation likely through electrostatic stabilization by a coordinated water or hydroxyl group (Jia et al., 2017).

More recently, mutational analysis of sclareol synthase from *S. sclarea* (Caniard et al., 2012; Schalk et al., 2012) identified a single asparagine residue, Asn431, located at the helix G break that impacts stereochemical control of product outcome. Switching Asn431 to glutamine reprogrammed the hydroxylation at C-13 from forming the native product 13*R*-sclareol to selectively producing its stereoisomer 13*S*-sclareol, thus highlighting the critical role of this amino acid on stereospecific water addition (Jia et al., 2018a). Collectively, these studies support a possibly critical role of the helix G hinge region in the catalytic control of product specificity in distinct diTPS and likely other class I TPS enzymes from ancestral *ent*-kaurene synthases.

Numerous studies also illuminated enzyme-specific active site residues with impact on class I catalysis in mono- and sesqui-TPSs. For example, alanine substitution of Asn338 in the *Salvia fructicosa* 1,8-cineole synthase generated an enlarged active site contour and redirected catalysis to effective conversion of the C_15_ FPP substrate to yield α-bergamotene, β-farnesene, and related sesquiterpenoid products, highlighting how minor alterations in the active cavity can enable the accommodation of different chain length substrates (Kampranis et al., 2007). In addition, a triad of active site residues impacting TPS capacity for generating cyclic products was discovered using comparative studies of *Artemisia annua* amorphadiene synthase and β-farnesene synthase that produce contrasting cyclic and linear products, respectively (Salmon et al., 2015). Large-scale site-directed mutagenesis studies of active site residues distinct between both enzymes revealed two central residue switches that activate cyclization in β-farnesene synthase (Tyr402Leu) or revert cyclization in the Tyr402Leu mutant (Val476Gly) (**Figure 6E**). Interestingly, a third mutation (Tyr430Ala) restored cyclization activity in the Val476Gly mutant background, illustrating that the ability to form a cyclic product is controlled by combinatorial effects of these active site positions. A growing body of knowledge exists on active site residues that contribute to different rearrangements of the initial cyclic carbocation intermediates in mono- and sesqui-terpenoid biosynthesis. For example, structure-guided mutagenesis of *Mentha spicata* limonene synthase, the key enzyme in the menthol production (Lange et al., 2011), identified two amino acids, His579 and Trp324, substitution of which led to premature neutralization of the carbocation intermediate to form both linear and cyclic monoterpenoids, including myrcene, linalool, and terpineol (Srividya et al., 2015) (**Figure 6F**). Similarly, reciprocal mutagenesis analyses of the mono-TPSs, Sitka spruce (*Picea sitchensis*) 3-carene synthase, and sabinene synthase associated with tree resistance against white pine weevil (Hall et al., 2011) revealed that two corresponding residues, 3-carene synthase Leu596, and sabinene synthase Phe596 located near the helix G break are critical for rearranging the central α-terpinyl^+^ carbocation toward 3-carene and sabinene, respectively (Roach et al., 2014).

The above examples and numerous related structure-function studies not covered within the scope of this review provide a mere glimpse into the plasticity of TPS catalysis, which relies on a largely non-polar active site with various possible carbocation rearrangements that enable the formation of myriad terpenoid structures with minimal investment in evolving new enzymes. However, despite these advances, our understanding of TPS mechanisms remains incomplete, thus limiting the ability to apply such knowledge for predicting the complex carbocation cascades underlying TPS activity and engineering desired enzyme functions. For instance, the taxonomic rather than functional relatedness of plant TPSs limits the use of phylogenic analyses for functional prediction (Chen et al., 2011; Zerbe and Bohlmann, 2015). Moreover, product re-direction through TPS mutagenesis as discussed above can be accompanied by a decrease in overall enzyme activity or additional byproducts resulting from a loss of steric control in the active site (Peters and Croteau, 2002; Pelot et al., 2016; Mafu et al., 2017). In this context, combining TPS structural analysis with quantum chemical calculations and molecular dynamic modeling approaches is advancing as a powerful tool kit to examine and predict TPS-mediated reaction cascades as discussed in more detail in several recent expert reviews (Tantillo, 2010; Tantillo, 2011; Gao et al., 2012; Major et al., 2014). For example, computational quantum chemical analyses have provided deeper insight into the inherent energy states driving terpene carbocation rearrangements and offer tools for predicting terpene pathways, as shown—for example, for predicting the often multi-product reactions catalyzed by sesquiterpene synthases (Isegawa et al., 2014). Likewise, structural studies combined with molecular dynamic modeling of TPSs has been successfully employed to predict the chemical space of possible carbocation rearrangements in mono-, di-, and tri-TPSs (Tian et al., 2014; Tian et al., 2016; Driller et al., 2018). Specifically, modeling of the catalytically relevant closed conformer of taxadiene synthase enabled important insight into the yet incompletely understood conformational changes of class I TPSs that contribute to the enzymes’ control over product outcome (Schrepfer et al., 2016). In addition, detailed insights into how individual active site residues impact taxadiene synthase catalysis was revealed using a combined quantum mechanics and free energy simulation approach (Ansbacher et al., 2018). Current challenges for such computational approaches, such as predicting the role of water in the active site and the termination of the carbocation *via* deprotonation or water capture require further attention, but can likely be addressed with increasing computing resources and available structural information on a broader range of TPSs.

## Functional Elaboration of the Terpene Scaffold

The vast majority of terpenoids feature multiple functional decorations of the TPS-derived hydrocarbon scaffold that critically contribute to the diverse bioactivities of the metabolite class (Pateraki et al., 2015; Bathe and Tissier, 2019). These tailoring reactions almost invariably are initiated by position-specific oxygenations. Although these reactions can be facilitated by TPSs as outlined above, the vast majority of terpene functional modifications are controlled by the large family of cytochrome P450 monooxygenases that function as versatile catalysts for a variety of monooxygenation reactions, as well as phenol-coupling reactions, oxidative rearrangements, and oxidative C–C bond cleavage in some cases (Mizutani and Sato, 2011; Banerjee and Hamberger, 2018). Given the vast diversity of P450-controlled metabolic bifurcations, their roles in terpenoid metabolism will be on briefly discussed here. For a more expansive overview, we refer the reader to a selection of expert reviews (Nelson and Werck-Reichhart, 2011; Pateraki et al., 2015; Banerjee and Hamberger, 2018; Bathe and Tissier, 2019).

The P450 superfamily has expanded far beyond the mid-sized TPS families observed in most plants studied thus far and comprises on average more than 200 genes in an individual plant genome with various functions in both general and specialized metabolisms. Among the 127 currently defined plant P450 families, only a handful have been shown to play major roles in terpenoid metabolism. Within the CYP85 clan, members of the CYP88A subfamily serve as *ent*-kaurenoic acid oxidases in GA biosynthesis (Nelson and Werck-Reichhart, 2011), whereas CYP725 and CYP720B enzymes are specific to gymnosperm species and catalyze hydroxylation and carboxylation reactions in the formation of taxol in species of *Taxus* and DRAs in *Pinaceae* species, respectively (Ro et al., 2005; Ro and Bohlmann, 2006; Rontein et al., 2008; Hamberger et al., 2011; Guerra-Bubb et al., 2012). More prominently, multiple families within the large CYP71 clan contribute to the various functional modifications of C_10_–C_20_ terpenoids (Hamberger and Bak, 2013). This includes members of the CYP701A subfamily that act as *ent*-kaurene oxidases in GA metabolism and, in several species, have been recruited through gene duplication and neo-functionalization for the formation of defensive specialized diterpenoids as exemplified in *Arabidopsis*, maize, and rice (Morrone et al., 2010; Wang et al., 2012b; Mafu et al., 2018). However, the majority of terpenoid-modifying P450s fall into the vast CYP71 and CYP76 families with numerous such enzymes having been characterized. Both P450 families are presumably evolutionary younger with the CYP76 family first occurring in cycads and *Ginkgo*, whereas the CYP71 family seemingly emerged with the onset of angiosperm evolution but is absent in nonseed plants (Nelson and Werck-Reichhart, 2011). Members of both families predominantly function as position-specific hydroxylases that catalyze (poly-)oxygenations of various mono-, sesqui-, and di-terpenoid scaffolds (Swaminathan et al., 2009; Ikezawa et al., 2011; Wu et al., 2011; Wang et al., 2012a; Diaz-Chavez et al., 2013; Guo et al., 2013; Ignea et al., 2016; Mao et al., 2016; Scheler et al., 2016; Mafu et al., 2018) but also alternate functions such as diterpenoid epoxidation and the formation of furan rings in mono- and di-terpenoid metabolisms have been demonstrated (Bertea et al., 2001; Heskes et al., 2018; Mafu et al., 2018). Notably, the first three-dimensional structure for a membrane-bound plant P450 (*S. miltiorrhiza* CYP76AH1) has been reported (Gu et al., 2019), providing resources to gain deeper mechanistic insight into the activity of diterpenoid-metabolic P450. In addition, recent P450 characterization studies expanded terpenoid-metabolic functions to other P450 families such as the gymnosperm-specific CYP750 family with a (+)-sabinene-3-oxidase (CYP750B1) from Western red cedar potentially involved in producing the anti-herbivory monoterpenoid thujone (Gesell et al., 2015), as well as members of the CYP726A subfamily from castor bean (*Ricinus communis*) that catalyze epoxidation and oxidation reactions converting macrocyclic casbene and neocembrene scaffolds in *Euphorbiaceae* species (King et al., 2014; Luo et al., 2016).

In addition to and often subsequent to the activity of P450s in the functional elaboration of terpene scaffolds, several other enzyme families contribute to the biosynthesis of bioactive terpenoids. This includes, but is not limited to, the function of 2-oxoglutarate/Fe(II)-dependent dioxygenases (2-ODDs) (Farrow and Facchini, 2014), for example, in GA phytohormone metabolism, as well as members of often large methyl-, glycosyl-, and acetyl-transferases (Bathe and Tissier, 2019).

## Concluding Remarks

Continued investigation of the evolutionary divergence and function of the TPS family will provide important knowledge of the still incompletely understood roles of terpenoids in mediating defensive and cooperative interactions with other organisms and the environment at large (Tholl, 2015). However, to address knowledge gaps and experimental limitations, research in several areas will be particularly important. Advances in the computational annotation and biochemical characterization of TPSs and P450 enzymes must continue in order to fully capitalize on rapidly expanding sequence resources across a broad range of reference and non-model species. Here, application of combinatorial functional studies in both microbial and plant hosts systems have proven to be a powerful tool to analyze modular terpenoid-metabolic networks comprised of multiple functionally distinct enzymes (Zerbe et al., 2013; Kitaoka et al., 2015; Andersen-Ranberg et al., 2016; Johnson et al., 2019a). Along with more efficient identification of new enzyme functions, continued structure-function studies will provide a deeper understanding of the functional diversity and molecular evolution of species-specific enzymes and pathways. Likewise, advanced quantum and molecular mechanics approaches for protein modeling and carbocation docking can utilize deeper structural insight to improve the precision of TPS functional prediction (Isegawa et al., 2014; Chow et al., 2015; Tian et al., 2016; O’Brien et al., 2018). Knowledge of terpenoid-metabolic genes, enzymes, and pathways will increasingly enable the investigation of terpenoid physiological functions *in planta* and under various environmental conditions. To this end, gene editing and transformation techniques applicable to a broader range of model and non-model species that produce species-specific blends of bioactive terpenoids will be critical (Wurtzel and Kutchan, 2016). Together, advanced genomic and biochemical tools and a deeper understanding of terpenoid biosynthesis and function have tremendous potential for harnessing the natural diversity of plant terpenoids for, for example, improving crop resistance and other quality traits and developing advanced protein and pathway engineering strategies for producing known and novel bioproducts.

## Author Contributions

PK and PZ jointly wrote the manuscript.

## Funding

Work in the laboratory of the authors has been funded by the NSF Plant-Biotic Interactions Program (grant# 1758976 to PZ), by the DOE Joint Genome Institute Community Science Program (grant# CSP2568 to PZ), and by the DOE Early Career Research Program (grant# DE-SC0019178 to PZ).

## Conflict of Interest

The authors declare that the research was conducted in the absence of any commercial or financial relationships that could be construed as a potential conflict of interest.
